# Synthesis, Antiproliferative Activity and Molecular Properties Predictions of Galloyl Derivatives

**DOI:** 10.3390/molecules20045360

**Published:** 2015-03-25

**Authors:** Marciane Maximo da Silva, Marina Comin, Thiago Santos Duarte, Mary Ann Foglio, João Ernesto de Carvalho, Maria do Carmo Vieira, Anelise Samara Nazari Formagio

**Affiliations:** 1 Faculdade de Ciências Biológicas e Ambientais, Universidade Federal da Grande Dourados, Rodovia Dourados—Itahum, Km 12, Dourados, 79.804-970 MS, Brazil; E-Mail: marcianemaximo@hotmail.com; 2 Faculdade de Ciências Exatas e Tecnologia, Universidade Federal da Grande Dourados, Rodovia Dourados—Itahum, Km 12, Dourados, 79.804-970 MS, Brazil; E-Mails: marina_comin@hotmail.com (M.C.); thiagosantos948@gmail.com (T.S.D.); 3 Centro Pluridisciplinar de Pesquisas Químicas, Biológicas e Agrícolas, Universidade Estadual de Campinas, 6171, Campinas, 13083-970 SP, Brazil; E-Mails: foglioma@cpqba.unicamp.br (M.A.F.); carvalho_je@yahoo.com.br (J.E.C.); 4 Faculdade de Ciências Agrárias, Universidade Federal da Grande Dourados, Rodovia Dourados—Itahum, Km 12, Dourados, 79.804-970 MS, Brazil; E-Mail: mariavieira@ufgd.edu.br

**Keywords:** galloyl, hydrazide, oxadiazole, antiproliferative activity, *in silico* study

## Abstract

The present study was designed to investigate the *in vitro* antiproliferative activity against ten human cancer cell lines of a series of galloyl derivatives bearing substituted-1,3,4-oxadiazole and carbohydrazide moieties. The compounds were also assessed in an *in silico* study of the absorption, distribution, metabolism and excretion (ADME) in the human body using Lipinski’s parameters, the topological polar surface area (TPSA) and percentage of absorption (%ABS). In general, the introduction of *N'*-(substituted)-arylidene galloyl hydrazides **4**–**8** showed a moderate antitumor activity, while the 2-methylthio- and 2-thioxo-1,3,4-oxadiazol-5-yl derivatives **9** and **10** led to increased inhibition of cancer cell proliferation. The precursor compound methyl gallate **2** and the intermediary galloyl hydrazide **3** showed greater antiproliferative activity with GI_50_ values < 5.54 µM against all human tumor cell lines tested. A higher inhibition effect against ovarian cancer (OVCAR-3) (GI_50_ = 0.05–5.98 µM) was also shown, with compounds **2**, **3**, **9** and **10** with GI_50_ ≤ 0.89 µM standing out in this respect. The *in silico* study revealed that the compounds showed good intestinal absorption.

## 1. Introduction

Gallic acid (3,4,5-trihydroxybenzoic acid) is a polyphenol that possesses a wide spectrum of important pharmacological properties. In particular, gallic acid affects several pharmacological and biochemical pathways and has strong antioxidant [1,2], anti-inflammatory [3], antimutagenic and anticancer properties [4,5,6], showing selective cytotoxicity against a variety of tumor cells and much less toxicity against normal cells [7,8,9,10,11,12,13,14,15].

Studies with a variety of synthetic galloyl derivatives have demonstrated the influence of substituents on cytotoxic activity, e.g., digalloylresveratrol shows induction of apoptosis and necrosis in HT-29 colon cancer cells and HL-60 human promyelocytic leukemia cells, and gallic acid esters show inhibition of cancer cell proliferation [14,16,17,18,19,20,21,22,23]. Appropriate substituents in the galloyl group positions could lead to more potent compounds.

The isolation of methyl gallate from the methanol extract of *Schinus terebinthifolius* leaves by our research group (data not shown) and the biological potential of the galloyl group directed us to develop derivatives as antitumor agents. Mannich bases of oxadiazoles and carbohydrazides possess important activities, including anticancer functions [24,25,26,27] and our prior studies showed that introducing 1,3,4-oxadiazole and carbohydrazide units at the C-3 position of the *β*-carboline derivatives led to significant antitumor action [28,29,30]. As such, we proposed the synthesis and *in vitro* antiproliferative activity evaluation of a series of galloyl derivatives bearing the substituted-1,3,4-oxadiazole and carbohydrazide moiety at position C-1, expecting that the incorporation of these substituent may improve the antitumor activities of the galloyl group. Herein, the synthesis of 2-methylthio- and 2-thioxo-1,3,4-oxadiazol-5-yl derivatives is reported for the first time. Additionally, an *in silico* study of the absorption, distribution, metabolism, and excretion (ADME) properties of the compounds was performed by investigating their match of Lipinski’s rules, topological polar surface area (TPSA) and percentage of absorption (%ABS). *In silico* ADME is currently used widely to determine whether it is possible for a drug candidate to reach its site of action.

## 2. Results and Discussion

### 2.1. Chemistry

**Scheme 1:**
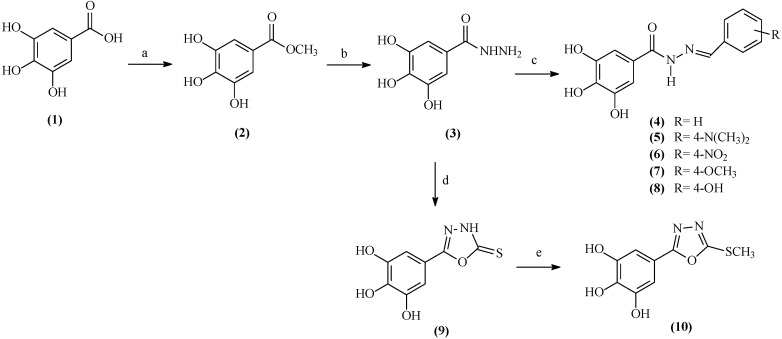
Synthetic route for the preparation of the galloyl derivatives.

The synthetic route for the preparation of the galloyl derivatives is presented in Scheme 1. Methyl gallate (**2**) was prepared by esterification of the corresponding carboxylic acid **1** with methanol and sulfuric acid [31]. The reaction of methyl gallate with hydrazine hydrate yielded the galloyl hydrazide **3**. Condensation of the hydrazide **3** with aromatic aldehydes (benzaldehyde, 4-dimethylaminobenzaldehyde, 4-nitrobenzaldehyde, 4-methoxybenzaldehyde and 4-hydroxybenzaldehyde), in ethanol heated at reflux, yielded the *N'*-(substituted)-arylidenegalloyl hydrazides **4**–**8**, respectively. The IR and ^1^H-NMR spectroscopic data of these compounds were consistent with the related literature and the experimental conditions and yields for the reaction were satisfactory [32].

*Reagents and conditions:* (a) MeOH, H_2_SO_4_, reflux, 48 h; 85%; (b) NH_2_NH_2_·H_2_O, EtOH, reflux, 48 h; 74%; (c) RCHO, EtOH, H_2_SO_4_ (cat), reflux, 36 h; 74%–80%; (d) CS_2_/KOH, EtOH, reflux, 48 h, acidified dilute HCl; (e) MeI, K_2_CO_3_, THF, rt, 48 h.

For preparation of new derivatives of galloyl-2-thioxo-1,3,4-oxadiazole (**9**), the key intermediate **3** was subjected to a reaction with carbon disulfide in the presence of KOH and ethanol heated at reflux. The 1,3,4-oxadiazolyl **9** was subsequently S-methylated with methyl iodide in the presence of K_2_CO_3_ at room temperature to yield afford the galloyl-2-methylthio-1,3,4-oxadiazole **10**.

The chemical structures of all compounds were confirmed by spectral data (^1^H- and ^13^C-NMR, IR, and MS) (see Section 3). The ^1^H-NMR spectra of *N'*-(substituted)-arylidene galloyl hydrazides **4**–**8** displayed signals at δ_H_ 8.40 ppm integrating for one proton and δ_H_ 6.70–9.00 ppm, corresponding to the imine and aromatic hydrogens, respectively, of the (substituted-benzylidene) galloyl hydrazide group. The presence of this group was confirmed by the signals at δ_C_ 147–150 (C=N), 158–162 (C=O), and 110–135 ppm (aromatic carbons of R group) in the ^13^C-NMR spectra.

The galloyl-2-thioxo-1,3,4-oxadiazole **9** may exist in a thione-thiol tautomeric structure, as indicated by the –NH and –SH proton signals at 13.7–14.1 ppm in the ^1^H-NMR spectrum, as a broad singlet integrating for one hydrogen each. In the solid state, these compounds are present in the thione (C=S) form, as indicated in their IR spectrum by the absence of the *ν*_s_(S–H) absorption band at 2500 cm^−1^ and the presence of two *ν*(C=S) absorption bands at 1230–1339 cm^−1^, which is characteristic for this class of compounds. The S-methylated derivative **10** was characterized by the presence of an addition signal at δ_H_/δ_C_ 2.84/14.7 ppm, corresponding to the S-methyl group attached to the oxadiazole nucleus.

### 2.2. Antiproliferative Activity

Three response parameters (GI_50_, TGI, and LC_50_) were calculated for each compound and cell line, and the results are summarized in Table 1 and Table 2.

**Table 1:** GI_50_ values (µM) for compounds **2**–**10** in ten tumor cell lines.

Cancer Cell Lines										
	U251	MCF-7	NCI/ADR-RES	786-0	NCI-H460	PC-3	OVCAR-3	HT-29	K-562	HaCaT
**2**	0.16	0.16	0.12	0.65	0.13	0.57	0.05	0.49	0.31	0.26
**3**	1.11	0.85	0.91	1.56	5.54	3.53	0.88	1.21	1.17	2.50
**4**	7.80	5.55	7.16	9.06	11.20	5.07	1.24	7.53	9.96	8.29
**5**	24.10	8.61	4.68	8.93	21.64	-	2.93	16.24	2.05	4.05
**6**	5.12	-	8.46	7.60	-	13.99	5.91	-	-	31.55
**7**	9.14	15.66	11.34	18.85	41.05	19.64	5.98	25.12	8.93	18.08
**8**	6.60	-	-	26.80	9.38	-	1.35	-	9.17	8.72
**9**	4.20	1.08	2.83	8.90	8.77	6.05	0.89	2.61	1.72	2.25
**10**	5.20	0.34	0.17	9.20	9.44	7.43	0.14	2.77	0.60	2.50
Dox	0.02	0.01	0.20	0.04	0.01	0.18	0.02	0.09	0.03	0.01

GI_50_: (growth inhibitory activity) the drug concentration that reduces cellular growth by 50%; Dox: Doxorubicin; (-): GI_50_ value not presented.

**Table 2:** TGI and LC_50_ (values in parentheses**)** in µg/mL for compounds **2**–**10** in ten tumor cell lines.

Cancer Cell Lines										
	U251	MCF-7	NCI/ADR-RES	786-0	NCI-H460	PC-3	OVCAR-3	HT-29	K-562	HaCaT
**2**	4.10	5.12	3.62	8.62	7.97	13.10	1.59	8.52	5.67	16.74
	(29.19)	(-)	(30.26)	(31.36)	(34.92)	(-)	(33.69)	(30.60)	(-)	(-)
**3**	6.13	2.36	2.56	17.98	17.35	18.31	9.77	3.27	-	29.02
	(31.36)	(-)	(-)	(-)	(-)	(-)	(-)	(26.21)	-	>100
**4**	-	23.54	-	-	-	-	29.61	-	-	-
		(-)					(-)			
**5**	-	20.26	83.07	-	-	-	41.52	48.50	8.98	18.41
		(47.93)	(-)				(-)	(-)	(-)	(55.32)
**6**	-	-	-	-	-	84.67	-	-	-	-
						(-)				
**7**	64.22	-	66.56	-	-	-	-	-	41.76	-
	(-)		(-)						(-)	
**8**	26.07	-	-	-	-	-	-	-	-	-
	(-)									
**9**	14.58	5.06	15.86	24.68	19.43	18.09	13.62	13.74	11.35	16.53
	(41.26)	(-)	(-)	(-)	(42.67)	(56.99)	(58.99)	(58.99)	(58.99)	(-)
**10**	16.40	3.20	2.33	29.40	29.52	28.45	2.38	17.66	3.70	18.00
	(30.43)	(20.24)	(19.65)	(-)	(65.78)	(69.30)	(17.34)	(60.32)	(20.20)	(-)
Dox	2.68	2.52	22.42	0.51	3.73	1.03	1.47	26.03	2.60	0.77
	(-)	(-)	(-)	(17.82)	(-)	(7.95)	(-)	(-)	(20.09)	(-)

TGI: the drug concentration required for total growth inhibition; LC_50_: the drug concentration required for killing 50% of cells; Dox: Doxorubicin; (-): not presented.

The GI_50_ values (growth inhibitory activity) (Table 1) refer to the drug concentration that produces a 50% reduction of cellular growth compared with the untreated control cells. The TGI (cytostatic activity) and LC_50_ (cytotoxic activity) values (Table 2) refer to the drug concentration required for total growth inhibition and killing 50% of the cells, respectively. GI_50_ values were used to classify a compound’s activity as follows: inactive, >100 µM; moderate, between >10 and <100 µM; and active, <10 µM.

The *N'*-(substituted)-arylidene galloyl hydrazide **4**, which has a phenyl group attached to the galloyl hydrazide moiety, possesses antiproliferative activity with GI_50_ values ranging from 1.24 to 9.96 µM for nine human tumor cell lines. In particular, the compound displayed activity against the ovarian cell line OVCAR-3 with a GI_50_ value of 1.24 µM (Table 1). Derivative **5**, which has a dimethylaminophenyl group attached to the galloyl hydrazide moiety, displayed cytotoxic efficacy against OVCAR-3 (GI_50_ = 2.93 µM; TGI = 41.52 µM) and leukemia K-562 (GI_50_ = 2.05 µM; TGI = 8.98 µM) cell lines (Table 1 and Table 2). Compounds **6** and **7** did not demonstrate cytotoxic efficacy in these cell lines. The activity against the OVCAR-3 cell line was also observed for compound **8** (GI_50_ = 1.35 µM), containing the *p*-hydroxyphenyl group to the galloyl hydrazide moiety.

The introduction of the 2-thioxo-1,3,4-oxadiazol-5-yl group **9** at C-1 of the galloyl group led to significant cytotoxicity, with GI_50_ less than 8.90 µM towards all cell lines, and displayed significant activity against OVCAR-3 (GI_50_ = 0.89 µM), MCF-7 (GI_50_ = 1.08 µM), K-562 (GI_50_ = 1.72 µM), HaCaT (GI_50_ = 2.25 µM), HT-29 (GI_50_ = 2.61 µM), and NCI/ADR/RES (GI_50_ = 2.83 µM) cell lines. The 2-methylthio analogue **10** showed an increase in antiproliferative activity, with particular effectiveness against OVCAR-3 (GI_50_ = 0.14 µM, TGI = 2.38 µM, LC_50_ = 17.34 µM), MCF-7 (GI_50_ = 0.34 µM, TGI = 3.20 µM, LC_50_ = 20.24 µM), K-562 (GI_50_ = 0.60 µM, TGI = 3.70 µM, LC_50_ = 20.20 µM) and NCI/ADR/RES (GI_50_ = 0.17 µM, TGI = 2.33 µM, LC_50_ = 19.65 µM) cell line (Table 1 and Table 2).

In addition, the precursor compound methyl gallate **2** exhibited potent, broad-spectrum antitumor activity with GI_50_ values < 1.0 µM against all human tumor cell lines tested, with GI_50_ (TGI) values ranging from 0.05 to 0.65 µM (1.59–16.74 µM) (Table 1 and Table 2). The intermediary, galloyl hydrazide **3**, also showed significant activity, with GI_50_ values in the range of 0.85–5.54 µM for all cell lines tested, and displayed cytotoxic efficacy against MCF-7 breast (GI_50_ = 0.85 µM, TGI = 2.36 µM), NCI/ADR-RES multiple drug resistant breast (GI_50_ = 0.91 µM, TGI = 2.56 µM) and OVCAR-3 ovarian (GI_50_ = 0.88 µM, TGI = 9.77 µM) cells (Table 1 and Table 2). Methyl gallate is a derivative of gallic acid that has been extensively investigated for important biological properties such as antioxidant, anti-inflammatory, antimicrobial and antitumor activities by inhibiting tumor infiltration of CD4^+^CD25^+^ regulatory T cell and glioma cells [19,33,34,35,36,37]. However, the antitumor activity of this compound has not been extensively examined in other cancer cells. We examined the effect of methyl gallate in ten human tumor cell lines and observed selective cytotoxicity in all cell lines tested.

An analysis of the relationship between the GI_50_ data and the effects of the substitutions in the aromatic ring at the *N'*-(substituted)-arylidene galloyl hydrazide with electron-withdrawing and electron-donating substituents **5**–**8** did not display enhanced activity compared with the phenyl substituent **4**, which demonstrated a pronounced effect on cell line proliferation. These results suggest that the electronic features of the various substituents affect how easily the drug can interact with biological molecules. Regarding the relationship between the 1,3,4-oxadiazolyl derivatives, compound **10** displayed the highest antiproliferative activity compared to the corresponding precursor **9**, exhibiting selectivity for four cell lines. Substitution by ester, hydrazine and 1,3,4-oxadiazolyl moiety side chains in compounds **2**, **3**, **9** and **10** resulted in a reduction in the percentage of growth inhibition, thereby indicating the importance of these side chain groups.

The anthracyclins doxorubicin (DOX) and daunorubicin (DNR) are among the most useful antibiotic, antitumor natural isolates from a species of the bacteria *Streptomyces* for the treatment of breast cancer, solid tumors and leukemia [38,39,40,41,42]. The mechanism of action is complex. Briefly, doxorubicin interferes with DNA replication by intercalation into DNA molecules, thus inhibiting the biosynthesis of DNA, RNA and protein as well as the induction of the free radicals inside the cell, leading to senescence and cell death by apoptosis or necrosis [43,44]. The compounds synthesized have functional groups similar to DOX, which suggests that they may act in a similar manner. Thus, the development of new molecular targets for antitumor therapies is directly correlated to many tumors that are resistant to therapeutic strategies and side effects. The main side effect of DOX is cardiac insufficiency, which is caused by the production of free radicals in the myocardium. In clinical research, chemotherapy resistance has been identified as a serious problem when the concentrations of chemotherapy drugs reach toxic and harmful doses for killing tumors.

### 2.3. Lipinski’s Rule of Five

The present study focused on the antiproliferative activity and synthesis of structurally modified galloyl derivatives, but we also explored the bioavailability of the synthesized derivatives, which is of importance for further development of drugs based upon these substances and their analogues. Drug-likeness is a promising paradigm to identify a balance that influences the pharmacodynamic and pharmacokinetic properties of a compound that ultimately optimizes its absorption, distribution, metabolism and excretion (ADME) in the human body [45].

These parameters were tentatively assessed using theoretical calculations following Lipinski’s rule of five, which establishes that the absorption or permeation of an orally administered compound is more likely to be efficient if the drug satisfies the established criteria: molecular weight (MW) ≤ 500 Da, log P ≤ 5, H-bond donors (HBD) ≤ 5 and H-bond acceptors (HBA).

Our results (Table 3) revealed that derivatives **2**, **4**–**10**, presented lipophilicities less than 5, with values between 0.34 and 1.94. Galloyl hydrazide (**3**) showed lipophilicity, Log P = −0.94, which is not in violation of Lipinski’s rules. To further evaluate the drug-likeness, the rules have spawned many extensions, such as the partition coefficient, Log P in −0.4 to +5.6 ranges [46]. All derivatives have a number of hydrogen bond acceptors (HBA) (*n*-ON = 5–9), and their molecular weights were smaller than 500 (184.15 > MW < 317.26), which is in agreement with Lipinski’s rules. With the exception of the hydrazide **3**, they all have a number of hydrogen bond donors (HBD) (*n*-OHNH = 6), violating one of the Lipinski’s rules. The calculated percent absorption (%ABS) of all derivatives ranged between 57.95% and 78.98%, indicating that these compounds have good permeability in the cellular membrane. Other rules include the number of rotatable bonds, indicating the flexibility of the molecule, the volume and the polar surface area. The topological polar surface area (TPSA) is recognized as a good indicator of drug absorption in the intestine (TPSA less than 140 Angstroms squared [Å^2^]) and blood-brain barrier penetration (TPSA less than 60 Å^2^). The compounds exhibit computational TPSA values between 86.99 and 122.37 Å^2^ and have good intestinal absorption except **6** (147.97 Å^2^). However, the derivatives do not have adequate blood-brain barrier penetration, as the TPSA values are more than 60 Å^2^.

The empirical conditions to satisfy Lipinski’s rule and to manifest a good oral bioavailability involve a balance between the solubility of a compound and its ability to diffuse passively through the different biological barriers. Compounds with high solubility are more easily metabolized and eliminated from the organism, thus leading to a lower probability of adverse effects and bioaccumulation. The compound **2** (Log S = −0.87) presented good solubility, thus encouraging its use as a precursor to produce new drugs that have better absorption.

**Table 3:** Lipinski’s rule and %ABS, TPSA, Log S for compounds **2**–**10**.

			Lipinski’s Parameters					
Comp.	%ABS	TPSA *^a^* (Å^2^)	nHBA *^a^* (nON)	nHBD *^a^* (nOHNH)	Log P *^a^*	MW *^a^*	n violations *^a^*	Log S *^b^*
**2**	78.98	86.99	5	3	0.85	184.15	0	−0.87
**3**	69.04	115.80	6	6	−0.94	184.15	1	−1.09
**4**	73.75	102.15	6	4	1.84	272.26	0	−2.83
**5**	72.64	105.38	7	4	1.94	315.33	0	−2.87
**6**	57.95	147.97	9	4	1.80	317.26	0	−3.47
**7**	70.57	111.38	7	4	1.89	302.29	0	−2.85
**8**	66.78	122.37	7	5	1.36	288.26	0	−2.54
**9**	73.63	102.51	6	4	0.34	226.21	0	−2.10
**10**	74.63	99.61	6	3	1.27	240.24	0	−3.07

*^a^* www.molinspiration.com [47]; *^b^* www.organic-chemistry.org/prog/peo [48]; %ABS = 109 − 0.345 × TPSA; Number hydrogen bond acceptor (NO) *= n*HBA ≤ 10; Number hydrogen bond donors (OHNH) = *n*HBD ≤ 5; MW ≤ 500; Octanol-water partition coefficient = Log P < 5; Solubility = Log S between −1 and −5.

## 3. Experimental Section

### 3.1. Chemistry

#### 3.1.1. General

^1^H- and ^13^C-NMR spectra were recorded at 300 MHz and 75 MHz, respectively, using a model Mercury plus BB spectrometer (Varian, Palo Alto, CA, USA). Mass spectra (MS) were recorded in a Focus-DSQ II spectrometer (Thermoelectron Corporation, Austin, TX, USA). IR spectra were recorded on a model MB-100 spectrometer (BOMEM, Quebec, CA, USA). For TLC, precoated plates (silica gel 60 G254, Macherey-Nagel, Duren, GER) were used. All reagents were purchased from commercial suppliers.

#### 3.1.2. Galloyl hydrazide (**3**)

Briefly, 80% hydrazine hydrate (1.8 mL, 48.2 mmol) was added to a solution of methyl gallate (**2**) (2.5 g, 2.97 mmol) that was previously prepared by esterification of commercial gallic acid (**1**) in ethanol (30 mL). This mixture was heated at reflux for 60 h, when TLC analysis indicated the end of the reaction. Then, the media was poured on ice, and the resulting precipitate was filtered, affording the title compound **3** (1.85 g, 74%) as pale white powder, mp 164–167 °C (MeOH); mp 167 °C [32]; IR (KBr) ν_max_ (cm^−1^): 3365–3284 (O-H, N-H), 1623 (C=O), 1509–1303 (C=C), 1260 (C-O); ^1^H-NMR (DMSO-*d*_6_): δ_H_ 6.77 (s, 2H); 8.59 (NH); 9.32 (OH). ^13^C-NMR (DMSO-*d*_6_): δ_C_ 106.5, 123.9, 136.2, 145.4, 166.4. MS *m/z* (%): 185 (MH^+^, 12), 153 (100), 126 (42).

#### 3.1.3. General Procedure for the Preparation of *N'*-(Substituted)-arylidene galloyl hydrazides **4**–**8**

A solution of galloyl hydrazide (**3**) (0.28 g, 1 mmol) in water (10 mL) containing two drops of conc. H_2_SO_4_ was heated at reflux for 0.5 h until complete dissolution. Then, aromatic aldehydes (benzaldehyde, 4-*N*-dimethylaminobenzaldehyde, 4-nitrobenzaldehyde, 4-methoxybenzaldehyde and 4-hydroxybenzaldehyde, 1.5 mmol) in EtOH (3 mL) were added to the solution, which was then heated at reflux for 48 h. The mixtures were poured into cold water and neutralized with 10% aqueous NaHCO_3_. The precipitates were collected by filtration, recrystallized and dried, yielding the compounds **4**–**8** in 74%–83% yields.

*N'-(Benzylidene)galloyl hydrazide* (**4**). White powder (0.20 g, 71%), mp 170–173 °C (MeOH); mp 170–172 °C [32]; IR (KBr) ν_max_ (cm^−1^): 3362–3270 (O-H, N-H), 1630 (C=O), 1518 (C=C), 1264 (C-O); ^1^H-NMR (CD_3_OD): δ_H_ 7.30–7.68 (5H, m), 7.70 (2H, s), 8.65 (1H, s, N'=CH). ^13^C-NMR (CD_3_OD): δ_C_ 115.4, 126.3, 128.7, 128.8, 131.0, 133.9, 140.4, 145.2, 148.7, 162.5. MS *m/z* (%): 272 (MH^+^, 8), 153 (100), 126 (30).

*N'-(4-Dimethylaminobenzylidene)galloyl hydrazide* (**5**). White powder, (0.22 g, 78%) mp 174–178 °C (MeOH); mp 175–178 °C [32]; IR (KBr) ν_max_ (cm^−1^): 3325 (O-H, N-H), 1661 (C=O), 1520 (C=C), 1264 (C-O); ^1^H-NMR (CD_3_OD): δ_H_ 3.03 (6H, s), 6.93 (2H, d, *J* = 8.7 Hz), 7.68 (2H, s), 7.78 (2H, d, *J* = 8.7 Hz), 8.36 (1H, s, N'=CH). ^13^C-NMR (CD_3_OD): δ_C_ 40.3, 111.9, 114.1, 125.9, 129.8, 130.4, 141.4, 146.2, 149.4, 151.2, 162.6. MS *m/z* (%): 315 (MH^+^, 12), 153 (100), 126 (33).

*N'-(4-Nitrobenzylidene)galloyl hydrazide* (**6**). Pale yellow powder (0.24 g, 83%); mp 171–174 °C (MeOH); mp 171–172 °C [32]; IR (KBr) ν_max_ (cm^−1^): 3422–3280 (O-H, N-H), 1665 (C=O), 1520 (C=C), 1264 (C-O); ^1^H-NMR (CD_3_OD): δ_H_ 6.98 (2H, s), 7.94 (2H, d, *J* = 8.5 Hz), 7.28 (2H, d, *J* = 8.5 Hz), 8.49 (1H, s, N'=CH), 9.19 (OH, NH). ^13^C-NMR (CD_3_OD): δ_C_ 116.3, 122.8, 124.1, 127.8, 137.3, 141.0, 143.9, 145.6, 147.6, 163.4; MS *m/z* (%): 317 (MH^+^, 10), 153 (100), 126 (21).

*N'-(4-Methoxybenzylidene)galloyl hydrazide* (**7**). Pale white powder (0,23 g, 80%), mp 170–172 °C (MeOH); mp 171–173 °C [32]; IR (KBr) ν_max_ (cm^−1^): 3410–3260 (O-H, N-H), 1668 (C=O), 1520 (C=C), 1262 (C-O); ^1^H-NMR (CD_3_OD): δ_H_ 3.87 (3H, s, OCH_3_), 7.00 (2H, s), 7.70 (2H, d, *J* = 8.3 Hz), 6.80 (2H, d, *J* = 8.3 Hz), 8.58 (1H, s, N'=CH). ^13^C-NMR (CD_3_OD): δ_C_ 55.6, 110.2, 114.4, 126.5, 128.6, 130.0, 136.8, 145.5, 146.6, 160.5, 161.6; MS *m/z* (%): 302 (MH^+^, 31), 153 (100), 126 (17).

*N'-(4-Hydroxybenzylidene)galloyl hydrazide* (**8**). White powder (0,20 g, 78%), mp 170–173 °C (MeOH); mp 172–174 °C [32]; IR (KBr) ν_max_ (cm^−1^): 3418–3266 (O-H, N-H), 1668 (C=O), 1571 (C=C), 1260 (C-O); ^1^H-NMR (CD_3_OD): δ_H_ 7.12 (2H, s), 7.34 (2H, d, *J* = 7.8 Hz), 6.60 (2H, d, *J* = 7.8 Hz), 8.66 (1H, s, N'=CH). ^13^C-NMR (CD_3_OD): δ_C_ 115.6, 114.8, 125.5, 128.7, 132.0, 136.8, 147.5, 147.6, 158.5, 160.6; MS *m/z* (%): 288 (MH^+^, 18), 153 (100), 126 (44).

#### 3.1.4. Galloyl-2-thioxo-1,3,4-oxadiazole (**9**)

Carbon disulfide (0.8 mL, 5 mmol) and potassium hydroxide (0,213 g, 1 mmol) were added to a solution of galloyl hydrazide (**3**) (0.456 g, 1 mmol) in EtOH (10 mL) at 0 °C. The resulting solution was heated at reflux for 48 h. The solvent was evaporated, and the residue was dissolved in water and acidified with a dilute solution of HCl. The solid obtained was filtered and recrystallized to give the title compound **9** (0.36 g, 80%) as a white powder, mp 168–170 °C (MeOH); IR (KBr) ν_max_ (cm^−1^): 3370–3280 (O-H), 1595–1430 (C=C), 1245 (C-S), 1168 (C-O-C); ^1^H-NMR (CD_3_OD): δ_H_ 6.82 (2H, s), 9.05 (OH, NH); ^13^C-NMR (CD_3_OD): δ_C_ 105.0, 112.0, 137.6, 146.5, 161.1, 176.9. MS *m/z* (%): 226 (MH^+^, 12), 150 (100), 77 (28).

#### 3.1.5. Galloyl-2-methylthio-1,3,4-oxadiazole (**10**)

To a solution of galloyl-2-thioxo-1,3,4-oxadiazole (**9**) (0.20 g, 0.6 mmol) in anhydrous THF (10 mL) was to added K_2_CO_3_ (0.14 g, 0.6 mmol). The resulting solution was stirred at room temperature for 1 h. Methyl iodide (0.19 mL, 0.8 mmol) was then added, and the mixture was stirred for an additional 48 h. The solvent was removed, and the residue obtained was crystallized to give the title compound **10** (0.1192 g, 60%), as a brown powder, mp 166–168 °C (MeOH); IR (KBr) ν_max_ (cm^−1^): 3376 (O-H, N-H), 1668 (C=O), 1565–1430 (C=C), 1170 (C-O-C), 720 (C-S-C); ^1^H-NMR (CD_3_OD): δ_H_ 2.80 (3H, s, SCH_3_); 7.00 (2H, s); 9.62 (OH). ^13^C-NMR (CD_3_OD): δ_C_ 16.2, 110.2, 112.4, 136.6, 146.8, 162.2, 160.4; MS *m/z* (%): 240 (MH^+^, 8), 150 (100), 91 (28).

### 3.2. Anticancer Assay in Vitro

The synthesized compounds were assessed in the following ten human tumor cell lines from various tissues kindly provide by the National Cancer Institute (Frederick, MD, USA): U251 (glioma, CNS), MCF-7 (breast), NCI-ADR/RES (breast expressing the multiple drug resistance phenotype), 786-0 (renal), NCI-H460 (lung, non-small cells), PC-3 (prostate), OVCAR-3 (ovarian), HT-29 (colon), K-562 (leukemia) and HaCaT (keratinocytes). Stock cultures were grown in media containing 5 mL of RPMI 1640 (Gibco BRL) supplemented with 5% fetal bovine serum. Gentamicin (50 mg/mL) was added to the experimental cultures. Cells in 96-well plates (100 µL cells/well) were exposed to sample concentrations in DMSO/RPMI (0.25, 2.5, 25, and 250 µg/mL) at 37 °C and 5% CO_2_ for 48 h. The final DMSO concentration did not affect cell viability. Next, cells were fixed with 50% trichloroacetic acid, and cell proliferation was determined by spectrophotometric quantification (540 nm) of cellular protein content employing the sulforhodamine B assay [49]. Doxorubicin (0.025–25 µg/mL) was used as a positive control. Three measurements were obtained: first at time point zero (T_0_, at the beginning of incubation) and then 48 h post-incubation for both the compound-free (C) and tested (T) cells. Cell proliferation was determined using the equation 100 × [(T − T_0_)/C − T_0_]. A cytostatic effect was observed when T ≥ T_0_, while a cytocidal effect occurred when T < T_0_. The experiments were performed in triplicate.

### 3.3. In Silico Study

An *in silico* computational study of the synthesized compounds **2**–**10** was performed to determine Lipinski’s rules, topological polar surface area (TPSA) and percentage of absorption (%ABS). Calculations were performed using Molinspiration online property calculation toolkit software [47] and OSIRIS property explorer software [48]. The percentage of absorption was estimated using the following equation: %ABS = 109 − [0.345 × TPSA]. Lipinski’s rule states that an orally active drug generally has no more than one violation of the following criteria [50]:

(I) hydrogen bond donors ≤ 5 (OH and NH groups);
(II) hydrogen bond acceptors ≤ 10 (N and O atoms);
(III) molecular weight < 500;
(IV) calculated logP < 5.

## 4. Conclusions

The results suggest that synthetic derivatives of *N'*-(substituted)-arylidenegalloyl hydrazide do not have a very strong synergistic effect in the inhibition of cancer cell proliferation compared with methyl gallate (**2**), galloyl hydrazide (**3**) and 1,3,4-oxadiazolyl derivatives **9** and **10**. These compounds showed higher inhibition effect against ovarian (OVCAR-3) cells. Computational drug-likeness and TPSA calculations revealed that the compounds show good intestinal absorption. Finally, the *in silico* study identified these derivatives as potential new drug candidates. More detailed studies with other models, such *in vivo* assays, are essential for the characterization of these derivatives as anticancer agents.
